# The Migration and Transformation of Heavy Metals in Sewage Sludge during Hydrothermal Carbonization Combined with Combustion

**DOI:** 10.1155/2018/1913848

**Published:** 2018-06-27

**Authors:** Meng Liu, Yufeng Duan, Kagiso Bikane, Liang Zhao

**Affiliations:** ^1^Key Laboratory of Energy Thermal Conversion and Control of Ministry of Education, Southeast University, Nanjing 210096, China; ^2^Department of Chemical Engineering, Imperial College London, SW7 2AZ, UK; ^3^College of Materials Science and Engineering, Nanjing Forestry University, Nanjing 210037, China

## Abstract

The migration and transformation behaviors of heavy metals (HMs), including Cr, Mn, Ni, Cu, Zn, As, Cd, and Pb, during the hydrothermal carbonization (HTC) of sewage sludge (SS) were investigated. The immobilization of HMs during the combustion of solid residual (SR) produced from HTC of SS was also analyzed. With increasing HTC temperature and residence time, the majority of HMs (except As) accumulated in the SR. The residual rate of As in the SR decreased from 73.95% to 56.74% when the residence time was increased from 1h to 3h and reduced significantly from 73.95% to 37.48% when the temperature increased from 220°C to 280°C, implying that numerous arsenic compounds dissolved into liquid phase products. Although the HTC process has a positive influence on the transformation of HMs from weakly bound fractions to the more stable fractions, the exchangeable and reducible fractions of Mn, Zn, As, and Cd in the SR were still high. In addition, the leached amounts of Zn and As were high (14.61 and 6.16 mg/kg, respectively) and showed a high leaching risk to the environment. An increase in HTC temperature and residence time led to an increase of the residual rate of HMs in the combustion residual of SR, implying that the HTC process promotes the stabilization of HMs in the combustion process.

## 1. Introduction

Sewage sludge (SS) is a great potential energy resource and has attracted wide attention as a subject of research. However, it contains high concentrations of pollutants, including pathogenic bacteria, heavy metals (HMs), and toxic organic compounds [1, 2]. Various sludge-to-energy technologies, such as fertilization, anaerobic digestion, carbonization, pyrolysis, gasification, and combustion, have been developed for the recovery of useful energy from SS [3–6]. From the energetic, economic, and environmental points of view, combustion or co-combustion of SS with other solid waste using present equipment is a viable technique of dealing with the sludge instead of land-filling disposal [7]. However, the SS must be dehydrated to improve the net energy input during the combustion process. The dehydration process requires substantial amounts of heat, thereby increasing the economic burden. Hydrothermal carbonization (HTC) is a process whereby sludge can be converted into high-density solid fuel under mild temperatures and pressures [8–10]. In addition, waste heat from the HTC process can be used to preheat the raw materials and improve the recovery of energy [11, 12]. Zhao et al. [11] demonstrated that, under mild conditions (200°C, 30 min), energy recovery from sludge via the HTC process is more than 50%, showing better performance than the mechanical dewatering technology. During the HTC process, copious amounts of toxicity organics are decomposed and the contents of S, N, and Cl are reduced. Moreover, some HMs are stabilized in the solid residual (SR) [2, 13–18]. Therefore, HTC of SS for high-density and clean solid fuel preparation is a promising technology.

Influences of the HTC reaction conditions, including reaction time, temperature, and dewatering time, on the physicochemical properties of solid, liquid, and gas phase products have been widely investigated to explore the HTC mechanism of SS [7–15]. Furthermore, the optimized operation conditions were also obtained from previous studies. In recent years, several researchers have studied the migration and transformation behaviors of HMs during the HTC of SS [2, 16–21]. They mainly focused on the three aspects of the transformation of HMs. (1) The redistribution of HMs in the solid and liquid phase products: after HTC process, most HMs in the SS accumulate in the SR whilst minute amounts of HMs are released into liquid phase products. Additionally, as the temperature is increased, the quantity of HMs released into liquid products increases due to the extraction and decomposition effects of organics and minerals materials. Shi et al. [17] demonstrated that the quantity of HMs released into liquid was only 1.3%, with Cr, Ni, and Cu constituting the largest portion of the released HMs at 200°C. However, Wang et al. [19] found that the quantity of HMs released into liquid phase products was lower than 19%. (2) The chemical fractions and speciation of HMs: although different HMs show different transformation behaviors of chemical fractions, the HTC process may have a positive effect on the migration of HMs from bioavailable fractions into the more stable fractions. Shi et al. [17] found that the concentrations of acid soluble/exchangeable and reducible fractions of HMs (Zn, Cd, Pb, Cr, Ni, and Cu) in the SR decreased whilst the residual fraction increased when the HTC temperature was increased. This suggested that the weaker bound fraction of HMs was transformed into the more stable bound fraction. However, Wang et al. [19] found that the acid soluble/exchangeable and reducible fractions of Zn, Ni, and Cd in the SR were high, presenting potential risk to the environment. (3) The changes in the leaching characteristics of HMs: after the HTC process, the leachable amounts and leaching rate of HMs were reduced, indicating a reduction of the leaching toxicity of HMs in the SR. However, the leaching percentages of Zn, Ni, and Cd in the SR were still high and showed a considerable risk to the environment [19]. From the aforementioned works, the HTC process reduces the toxicity and improves the stabilization of HMs in the SR, but the transformation behaviors of HMs is significantly different, even some HMs still pose a high risk to the environment after HTC process. Considering the difference in the properties of SS and the effect of operation conditions on the transformation of HMs, the detailed mechanism of HTC influences the distribution and chemical speciation transformation of HMs in the SS requires further investigation.

Combustion of SR produced from the HTC of SS is considered a promising approach to convert sludge to energy, particularly the cocombustion with other solid fuels [22–24]. After the HTC process, SR showed a lower activation energy and preexponential factor. More than 60% of nitrogen and sulfur within SS can be removed, making it suitable for use in the combustion equipment [14, 22]. The transformation and immobilization behaviors of HMs during the combustion of SR produced from HTC of SS are crucial for the reduction of HMs emissions in the combustion process. However, no related studies were reported. Researchers [19] have only investigated the transformation behaviors of HMs in the pyrolysis of SR produced from HTC of SS. They found that the ecotoxicity of HMs in the pyrolysis residual reduced and the HMs could be migrated from bioavailable fractions to the more stable fractions.

In this work, the HTC reaction temperatures and residence times are considered systematically to investigate the distribution and chemical speciation transformation behaviors of HMs in the HTC of SS. Additionally, the immobilization characteristics of HMs during the combustion of SR produced from HTC of SS were analyzed.

## 2. Experimental

### 2.1. Materials

Dewatered municipal SS with the moisture content of 84.5 wt.% was obtained from a wastewater treatment plant in Nanjing, China. All the samples were sealed in a glass beaker and kept in a refrigerator at 4°C. The fuel characteristics of air dry basis raw SS are shown in Table 1. Raw SS has a high ash content (46.9 wt.%), low fixed carbon content (3.9wt.%), and an HHV (10.11kJ/kg). Additionally, the elemental contents of C, H, N, S, and O are 20.13, 4.16, 4.68, 1.73, and 12.41 wt.%, respectively.

### 2.2. Experiment Procedure of HTC

**Figure 1:**
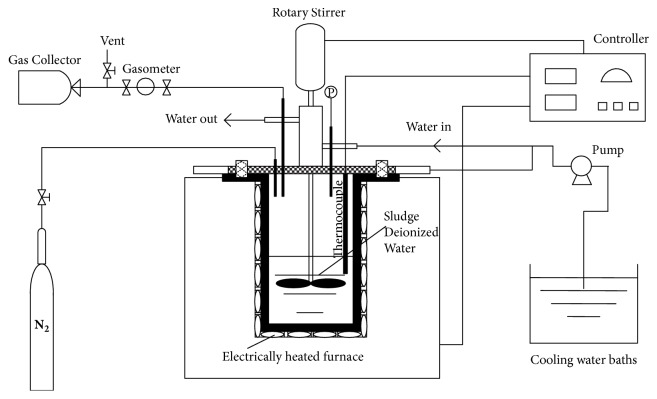
Schematic figure of hydrothermal batch-type reactor.

All HTC experiments were carried out in a batch-type reactor with a volume of 1 L. The schematic of reactor is shown in Figure 1. The reactor was heated by an electric furnace and the reaction temperature was controlled by a PID control unit. During each experiment, 500 g of SS and 100 g of deionized water were fed to the reactor, and the air in the reactor was discharged using high-pressure pure nitrogen. The reaction temperature (i.e., 220, 250, and 280°C) was attained at a heating rate of 5°C/min and the residence times investigated were 1, 2, and 3 h, respectively. The mixer was stirred at 700 rpm to maintain the homogeneity of the reaction. After the reaction, the reactor was rapidly cooled to room temperature using running water. The waste liquid (WL) and SR were collected from the reactor and subsequently separated by suction filtration. When the filtration step was complete, the SR was dried at 105°C for 2 h and ground into fine particles (less than 200 *μ*m) for further analysis. The WL was then placed in a refrigerator at 4°C. All HTC experiments were carried out three times to ensure the accuracy of data. Operating conditions were labeled using a format of “SS-xxx-xx”, where “xxx” is the reaction temperature and “xx” is the reaction time. For example, “SS-220-1h” represents the HTC of SS at 220°C for 1 h.

### 2.3. Fractionation Procedure of HMs

Speciation of HMs in SS and SR was investigated using the three-step BCR sequential extraction procedure [26]. Four fractions of HMs were extracted, including an acid soluble/exchangeable fraction (F1), a reducible fraction (F2), an oxidizable fraction (F3), and the residual fraction (F4). Detailed steps of the BCR method are reported in the previous studies [26]. For F1~F3, the suspension was collected after the centrifugation step at 10000 rpm for 20 min and diluted to a constant volume with 2% HNO_3_. Fine particles were removed by filtration and dissolved organics were degraded by digestion using H_2_O_2_ and concentrated HNO_3_. The F4 fraction and the total amounts of HMs in SS and SR were extracted by digestion with aqua regia solution and subsequently heated on a hot plate. All digestion solutions were filtered and stored in a refrigerator at 4°C prior to ICP-MS analysis.

### 2.4. Leaching Test

The toxicity characteristic leaching procedure (TCLP) has been widely used to evaluate the leach ability of HMs in the SS and SR [27]. TCLP leachates of SR were conditioned using the acetic acid solution (pH 2.88, liquid/solid ratio=20:1) and were subsequently shook at 200 rpm for 18 h. After centrifugation and filtration, the samples were digested with H_2_O_2_/HNO_3_ to remove dissolved organics.

### 2.5. Analysis

The ultimate and proximate analysis of the SS and SR were determined using Elemental Analyzer (LECO-CHNS 932, USA) and an Infrared Rapid Analyzer (5E-MACIII, China), respectively. The samples' calorific value was determined using the adiabatic bomb calorimetric method. The HMs content was measured using an ICP-MS (PerkinElmer Elan 9000, LabX, Canada). The composition of elements and minerals in the SS and SR was analyzed by XRF (ARL QUANTX, Thermo Fisher, USA) and XRD (Smartlab 3, Japan), respectively. The ammonia nitrogen in the WL was analyzed with a continuous flow analyzer (AutoAnalyzer3, SEAL).

### 2.6. Combustion Condition

The combustion process of SS and SR was conducted in a Thermo Gravimetric Analyzer (SETSYS-1750CSEvol, France). The reaction temperature and heating rate were set at 900°C and 20°C/min, respectively. O_2_/N_2_ (volume 1:4) was used to simulate air atmosphere, and the total flow rate was 50 ml/min. The mass of solid samples was accurately kept at 10 mg for each experiment. The combustion residual was collected for digestion using HCl, HNO_3_, HF, and HClO_4_, successively. After digestion, the liquid samples were sent to an ICP-MS to measure the content of HMs. Each combustion experiment was carried out three times to obtain accuracy data.

## 3. Results and Discussion

### 3.1. Physicochemical Properties of SR after HTC

Table 1 shows the physicochemical properties of SS before and after HTC. After the HTC process, the moisture content and volatile matter reduced significantly whilst the fixed carbon and ash content increased, implying that the HTC process has a positive effect on the fuel characteristics of SS. In addition, the moisture and oxygen content of SR reduced from 1.95 wt.% and 8.83 wt.% to 1.34 wt.% and 0.35 wt.%, respectively, when the temperature was increased from 220 to 280°C. This suggests that the dehydration of sludge significantly occurs during the HTC process. The characteristics presented above led to a slight increase of the HHV of SR. After filtration and before drying, the moisture of SR reduced from 64.9 wt% to 48.5% when the reaction temperature was increased from 220 to 280°C. This observation is associated with the decomposition of protein, polysaccharide, and other macromolecule organic compounds [25, 28]. Moreover, an increase in the reaction temperature from 220 to 280°C led to an increase of the WL pH value from 8.7 to 9.5. During the HTC of SS, the concentration of ammonium nitrogen organics in the WL increased significantly when the reaction temperature increased. This was attributed to the breakage of N-containing functional groups [29]. This is consistent with the concentration of NH_4_^+^-N in the WL shown in Table 1. It can be observed that the concentration of NH_4_^+^-N in the WL increased significantly from 1734 to 2245 mg/L when the temperature was increased from 220 to 280°C. Based on the results of Table 1, the influence of reaction temperature on the fuel quality improvement of HTC of SS is greater than that of the reaction residence time.

### 3.2. HMs Concentrations and Redistribution in the SR and WL

**Figure 2:**
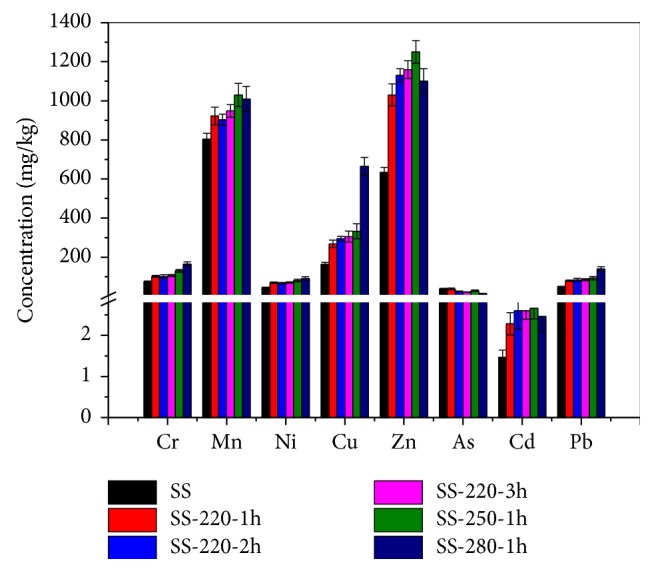
Concentrations of HMs in SS and SR.

It is well known that hydrothermal treatment, including hydrothermal carbonization [17–19], hydrothermal liquefaction [30–32], and hydrothermal gasification [33], has a significant effect on the transformation of HMs in the SS. The total concentrations of Cr, Mn, Ni, Cu, Zn, As, Cd, and Pb in the SS and SR are shown in Figure 2. As seen from Figure 2, the considerable amounts of Mn and Zn were present; however, relatively low concentrations of Cr, Cu, As, and Pb were observed in the SS. After the HTC process, nearly all the HMs concentrations increased with an increase in reaction temperature and residence time, particularly for Mn and Zn. In addition, there was a significant increase for Cu at 280°C. However, the concentration of As decreased slightly with an increase in temperature and residence time, suggesting that some of the arsenic compounds were dissolved into liquid phase products after the HTC process. From Figure 2, HMs seems to be effectively accumulated in the SR after the HTC process, which is closely related to the physical structures of heavy metal crystal and the condition of HMs under certain temperatures and pressures.

Since the HTC of sludge is always conducted at low temperature (less than 350°C), the concentration of HMs in the gas phase products is extremely low and is usually not analyzed. The transformation and stabilization effects of HMs exist simultaneously during the HTC process, resulting in the redistribution of HMs in the solid and liquid phase products. The residual rate of HMs is a parameter that determines the distribution behaviors of HMs during the HTC or combustion process. It can be calculated using the flowing equation:

(1) $$\begin{array}{c} R_{c}=\frac{{C2}_{x}\times m_{2}}{{C1}_{x}\times m_{1}}\times 100\% \end{array}$$

where* R*c is the residual rate of HMs in the HTC/combustion SR; x is the type of heavy metal; *C*2_x_ is the total concentration of x in the SR; *m*_2_ is the mass of solid residual (kg); *C*1_x_ is the total concentration of x in the raw samples (mg/kg); *m*_1_ is the mass of raw samples (kg).

The residual rate of HMs in the SR and WL is presented in Table 2. The results indicate that HTC process seems to have some positive effect on the release of HMs from the solid into liquid phase products. This might be due to the decomposition of extracellular polymeric substances, resulting in breakage of weaker bonded of HMs [17, 19, 21]. As seen from Table 2, different HMs show different released behaviors based on the reaction temperature and residence time. The residual rates of HMs in the SR products, Mn, Ni, Zn, and As, reduced with an increase in the reaction temperature and residence time. The residual rate of As in the SR decreased significantly from 73.95% to 37.48% when the temperature was increased from 220 to 280°C and reduced from 73.95% to 56.74% when the residence time was increased from 1h to 3h. This indicated that many arsenic compounds were released into the liquid phase products and posed a substantial risk to the environment. The effect of reaction temperature on the dissolution of HMs into liquid phase products is greater than that of residence time. In general, elevated temperature can enhance the extraction effects of HMs and improve the degradation and transformation of organic compounds and minerals, resulting in an increase in the release rate of HMs from solid into liquid phase products [21, 34]. However, the residual rate of Cu and Pb in the SR increased from 90.10% and 97.46% to 99.62% and 99.19% when the temperature was increased from 220°C to 280°C. During the HTC process, Cu prefers to be bound to organic matter and form the Cu-sulfide substance with high stability [17, 19, 20, 35–37]. In addition, Pb is easily combined with the primary minerals, such as Ca, Mg, and Fe, through the ion exchange [38, 39]. In the case of Cr and Cd, the temperature and residence time have a different effect on the residual rate in the SR. When the temperature was increased from 220°C to 280°C, the residual rate of Cr was enhanced from 87.12% to 94.51% whilst that of Cd reduced from 80.88% to 66.40%. In addition, as the residence time increased from 1h to 3h, the residual rate of Cr reduced from 87.12% to 83.42%, but that of Cd increased from 80.88% to 84.51%.

**Figure 3:**
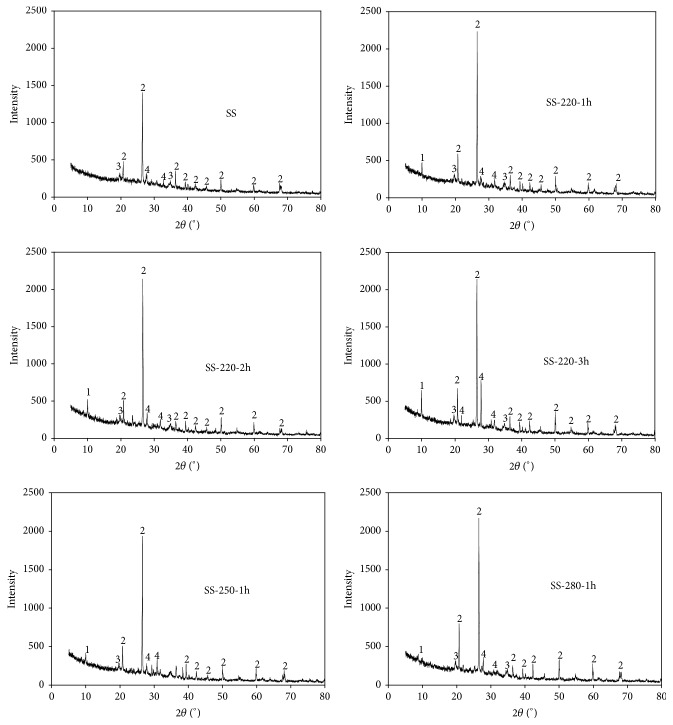
Minerals compositions of SS and SR. 1: K_2_Cu_3_+2O(SO_4_)_3_; 2: SiO_2_; 3: KAl_2_Si_3_AlO_10_(OH)_2_; 4: AlSO_4_(OH)·5H_2_O.

As described above, the minerals in the SS have a great influence on the absorption, precipitation, and complexation of HMs. Therefore, the minerals compositions of SS before and after the HTC process need to be investigated. The experimental results are shown in Figure 3. It can be deducted that the SS and SR were mainly composed of crystalline compounds, particularly Si, Cu, and Al compounds. After the HTC process, the intensity at 20° to 35° increased significantly, indicating that the crystalline phenomenon of the HMs increased. In addition, the HMs in the SR after the HTC of SS is mainly in the form of complex compounds, and parts of their crystal structures have a resemblance [40]. According to the literature [21, 41, 42], Fe/Al oxide and some inorganic minerals (clay, carbonate and phosphate, et al.) can combine and absorb the HMs through ion exchange and the surface complexation. Additionally, some organic functional groups (carboxyl and phenolic groups) and S, N-containing groups can reduce the migration of HMs in the SR via the complexation and adsorption [43]. The transformation and immobilization behaviors of HMs during the HTC process can be associated with some complicated physical-chemical processes, such as adsorption, precipitation complexation, and recombination, occurring between the HMs and the crystal lattices of SR [21, 36, 37, 44, 45]. However, detailed mechanisms of how the hydrothermal treatment affects the migration and transformation of HMs in sludge is extremely complex and still needs further investigation.

### 3.3. Fractions of HMs and Environment Risk Analysis of SR

#### 3.3.1. Fractions and Migration Behavior of HMs during the HTC Process

**Figure 4:**
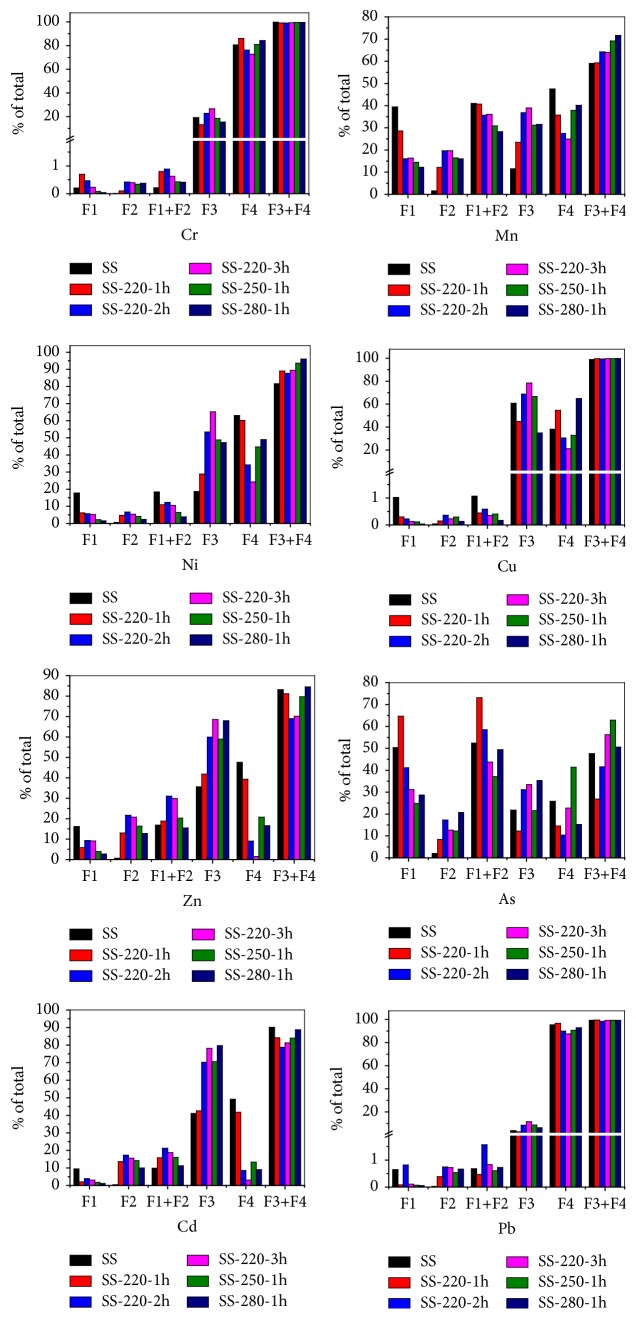
Speciation transformation behaviors of HMs in the SS before and after HTC.

Evaluation of environmental ecotoxicity of HMs predominantly depends on the chemical speciation of HMs. With reference to the bioavailability and ecotoxicity of HMs, F1 and F2 are identified as directly toxic fraction; F3 and F4 are considered as potentially toxic and nontoxic fractions, respectively. The transformation behaviors of the chemical speciation of HMs in the SS and SR after the HTC process are shown in Figure 4. The main fractions of HMs in the SS were found to be very different. Cr and Pb were mainly present in the F4 fraction (80.7% and 95.45%). More than 60% Ni and Cu were in the F3 and F4 fractions, respectively. The F1 fraction of Ni, Zn, and Cd was 17.7%, 16.2%, and 9.43%, respectively, whilst they almost have no F2 fraction. Mn and As had the high concentrations in the F1 fraction, with As having more than 50% of the total, implying that there exists high potential bioavailability and ecotoxicity. From Figure 4, Cr, Cu, and Pb are exceptionally low at the exchangeable (F1 <5%) fraction. Ni, Zn, and Cd are also low at the exchangeable (F1 <20%) fraction. However, Mn and As are high at the exchangeable (F1>40%) fraction.

At longer HTC residence times and higher temperatures, Cr and Pb showed no obvious changes in the F1 and F2 fraction but had a minute increase in the F3 fraction and slight decrease in the F4 fraction. Although Cu has a similar trend to Cr and Pb with regard to the change of F1 and F2 fractions, the F3 fraction of Cu sharply increased with residence time. There was also an observed initial increase followed a decrease when the temperature was increased from 220 to 280°C. An opposite trend was observed in the F4 fraction of Cu, indicating that the increase of the F3 fraction of Cu was mainly a result of migration from the F4 fraction. The Fe oxides composition favors the oxidation of copper and the combination of organic matters, resulting in the formation of Cu-oxide complexes during the HTC process [17, 19, 20, 35–37]. In the case of Mn, the HTC process showed a positive effect on the reduction of the F1 fraction when reaction temperature and residence time were increased, resulting in an increase in the F2 fraction. This suggests that the F1 fraction was converted into the F2 fraction. However, the directly toxic fractions (F1+F2) reduced with an increase in the reaction temperature and residence time. The percentage of Mn increased in the F3 fraction with increasing temperature and residence time. Contrastingly, it reduced in the F4 fraction with increasing residence time, indicating that some of the F4 fraction of Mn was converted into the F3 fraction and increased the potential bioavailability and ecotoxicity. Similar transformation behaviors of F1~F4 fraction were observed for Ni.

As for Zn and Cd, their F1 fractions reduced significantly whilst their F2 and F1+F2 fractions considerably decreased. The HMs F1+F2 fractions increased with the reaction residence time. When the temperature was increased from 220 to 280°C, there was an observed initial increase superseded by a reduction. The Zn and Cd F3 fractions increased whilst their F4 fraction reduced with an increase in the reaction residence time and temperature, indicating that most of the F4 fraction was converted to the F3 fraction. In addition, the F3+F4 fractions reduced significantly with an increase of the residence time. However, no pronounced changes with temperature were observed. From the results of Zn and Cd, the bioavailability and ecotoxicity increased after the HTC process, and the effect of residence time is greater than that of the reaction temperature. For As, the highest F1 fraction (64.6%) in the SR was obtained during the HTC of SS at 220°C for a residence time of 1h. Its F1 fraction reduced whilst its F2 fraction increased with an increase in the residence time and temperature. The F1+F2 fraction of As reduced with an increase in the residence time. With respect to increases in temperature, there was an observed increase in the F1+F2 fraction followed by a reduction. The As F3 fraction increased with residence time and temperature. These results indicate that there was practically no positive effect on the speciation transformation of As during the HTC process.

Basing on the preceding results, the weakly bound exchangeable fraction (F1) for HMs can be converted into F2 fraction as a result of the HTC process. However, HMs (Cr, Mn, Ni, Zn, Cd, and Pb) underwent a transformation from F4 fractions into F3 fractions. The F1+F2 fractions for Mn, Ni and Cu significantly reduced whilst those for Cr, Zn, As, and Cd increased. The F1 and F2 fractions of Mn, Zn, As, and Cd in the SR were high, suggesting a high potential bioavailability and ecotoxicity to the environment. As such, these results indicate that the HTC process has a positive effect on the chemical speciation transformation of HMs from weakly bound fractions to the more stable fractions.

#### 3.3.2. Leaching Characterization of SR

**Figure 5:**
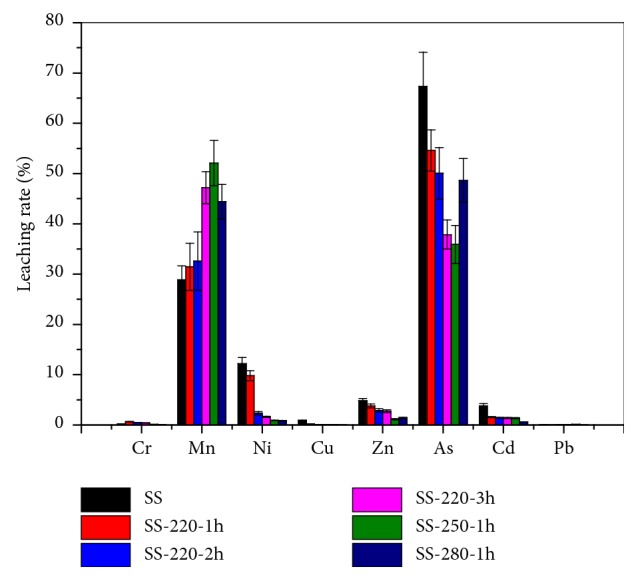
Leaching rates of HMs in the TCLP tests.

The leaching characteristics of HMs in the SS and SR were determined using the TLCP method [27]. The results are shown in Table 3 and Figure 5. The leached amounts of HMs from SS were 0.11, 231, 5.41, 1.42, 30.32, 25.57, 0.05 and 0.03 mg/kg for Cr, Mn, Ni, Cu, Zn, As, Cd and Pb, respectively. The leached amounts of Ni, Zn and As were higher than the permissible limits (USEPA, SW-846) and therefore exhibited potential toxicity. When the reaction temperature and residence time were increased, the leached amounts of Ni, Cu, Zn and As reduced significantly. However, the amount of Mn highly increased and no obvious changes were observed for Cr, Cd and Pb. In addition, the concentrations of leachable HMs in SS-220-3h were higher than those in SS-280-1h, indicating that the influence of reaction temperature on the reduction of leachable HMs was greater than that of residence time. Higher reaction temperatures contribute to the combination of HMs with crystal lattices of SR and enhance the immobilization of HMs [21, 40, 44, 45]. Briefly, the HTC process exhibited a significant reduction of the leaching risk of HMs. In contrast, the leached amounts of Zn and As were still high (14.61mg/kg and 6.16 mg/kg, respectively) and therefore showed a high leaching risk to the environment.

The leaching rate of HMs from SS and SR are shown in Figure 5. The leaching rate is defined as the ratio of the amount of a heavy metal in the leachate to the total amount of this heavy metal [17, 19, 46].

With an increase in the reaction temperature and residence time, the leaching rate of all HMs (except Mn and As) in the SR decreased significantly and almost exhibited no leaching rate. The leaching rate of As reduced when residence time was increased. However, the leaching rate of As first reduced and then increased when the temperature was increased from 220 to 280°C. The As leaching rate was close 50% at a reaction temperature of 280°C for a residence time of 1h. This exhibits a high leaching risk to the environment. As shown in Table 3 and Figure 5, the leachable fractions of HMs in the SR endured a significant reduction as a result of the HTC process. This observation however does not include As and Mn which had high leaching rates.

### 3.4. Immobilization Behaviors of HMs in the Combustion Residual

The immobilization behaviors of HMs during the combustion of SR produced from the HTC of SS were analyzed. The concentrations of HMs in the combustion residual are shown in Table 4. Compared to the combustion of SS, the respective amounts of HMs (except As) in the combustion residual of SR increased. In addition, the amounts of HMs in the combustion residual are higher than that in the SS and SR (Figure 2). This indicates that most of HMs accumulated in the combustion residual. However, as for As, the concentration changed considerably from 62.58 mg/kg to 38.39 mg/kg when the HTC SR changed from “SS-220-1h” to “SS-280-1h”. Arsenic has a low boiling point and therefore exhibits volatile and gasification properties [47]. Consequently, it can easily be gasified during the combustion process. In addition, some inorganic compounds (Ca, Al, and Fe compounds) can provide chemical reactive sites of arsenic and enhance the complexation and absorption of arsenic by forming various arsenates in the fly ash and solid residual [48–51]. From Table 4, the concentrations of HMs in the combustion residual have minimal pronounced changes even when the HTC reaction temperature and residence time were varied. Although the HTC process was carried out at different reaction conditions, the chemical compositions of SR tend to be stable and the crystal lattices of HMs are similar [40]. The residual rates of HMs after the combustion of SR produced from the HTC of SS are presented in Table 5. As seen from Table 5, the residual rates of all HMs were more than 70%, particularly for Cr, Ni, Zn, and Cd which had more than 90% residual rates. With increasing HTC reaction temperature and residence time, the residual rates of HMs (except Pb) in the combustion residual of SR increased, indicating that the HTC process promotes the immobilization of HMs in the combustion solid residual.

## 4. Conclusions

The transformation behaviors of Cr, Mn, Ni, Cu, Zn, As, Cd, and Pb in the SS during the HTC process combined with combustion were investigated. Most of the HMs accumulated in the SR during HTC and combustion process, respectively. However, the concentration and residual rate of As in the SR reduced with an increase in the HTC reaction temperature and residence time, implying that many arsenic compounds are released into the liquid phase products. After the HTC process, the weakly bonded fractions of HMs migrated to the more stable fraction. However, the exchangeable and reducible fractions of Mn, Zn, As, and Cd in SR were remarkably high and presented an elevated risk to the environment. The leaching characteristics of HMs in the SR showed a significant improvement. Contrastingly, the leached amounts of Zn and As were still high and exceeded the permissible limits. The contents of HMs in the combustion residual were higher than those in the SS and SR. The residual rates of almost all the HMs in the combustion residual of SR produced from the HTC of SS increased with increasing HTC temperature and residence time, indicating that the HTC process promotes the immobilization of HMs in the combustion process.

## Figures and Tables

**Table 1:** Physicochemical characteristic of SS before and after HTC.

Samples	Ultimate analysis/wt.%					Proximate analysis/wt.%				HHV^2)^	Moisture content of SR^3)^ (wt.%)	pH (WL)	NH_4_^+^-N (mg/L) (WL)
	C_ad_	H_ad_	N_ad_	S_ad_	O_ad_^1)^	M_ad_	V_ad_	FC_ad_	A_ad_	(kJ/kg)			
SS	20.13	4.61	4.68	1.73	12.41	9.54	39.66	3.90	46.90	10.11	-	ND	ND
SS-220-1h	23.87	3.45	3.49	1.12	7.71	1.95	32.74	6.90	58.41	10.31	64.90	8.70	1734
SS-220-2h	23.21	3.06	2.95	1.44	5.57	1.86	28.86	7.37	61.91	9.78	62.50	8.90	1773
SS-220-3h	23.65	3.14	2.82	1.82	5.28	1.59	29.37	7.34	61.70	10.06	59.35	9.00	1825
SS-250-1h	25.26	2.93	2.32	1.36	1.85	1.54	26.61	7.11	64.74	10.67	52.30	9.20	2005
SS-280-1h	26.69	2.64	2.16	0.87	0.28	1.34	25.07	7.47	66.02	11.28	48.50	9.50	2245

^1)^O(%)=100%-(C%+N%+H%+S+%M%+A%); ad-air dry basis.

^2)^HHV, higher heating value. Calculated was according to Channiwala and Parikh [25].

^3)^Moisture content of SR after filtration and before drying.

**Table 2:** Residual rates of HMs in the SR and WL after HTC process.

Samples	Percentage (%)															
	Cr		Mn		Ni		Cu		Zn		As		Cd		Pb	
	SR	WL	SR	WL	SR	WL	SR	WL	SR	WL	SR	WL	SR	WL	SR	WL
SR-220-1h	87.12	12.88	86.10	13.90	99.70	0.30	90.10	9.90	95.53	4.47	73.95	26.05	80.88	19.12	97.46	2.54
SR-220-2h	81.71	18.29	80.08	19.92	91.48	8.52	94.16	5.84	90.27	9.73	63.78	36.22	86.68	13.32	97.62	2.38
SR-220-3h	83.42	16.58	81.83	18.17	93.79	6.21	94.89	5.11	90.30	9.70	56.74	43.26	84.51	15.49	97.94	2.06
SR-250-1h	91.29	8.71	79.26	20.74	95.19	4.81	92.00	8.00	80.17	19.83	46.83	53.17	77.79	22.21	99.06	0.94
SR-280-1h	94.51	5.49	71.93	28.07	93.44	6.56	99.62	0.38	85.29	14.71	37.48	62.52	66.40	33.60	99.19	0.81

**Table 3:** Concentrations of leachable HMs in the TCLP tests (mg/kg).

HMs	Raw SS	SS-220-1h	SS-220-2h	SS-220-3h	SS-250-1h	SS-280-1h	Permissible limits^a^
Cr	0.11±0.00	0.61±0.01	0.44±0.01	0.39±0.01	0.09±0.00	0.09±0.00	5.0
Mn	231.06±10.67	289.22±10.56	293.71±12.45	446.73±16.56	536.53±16.78	449.39±14.34	---^b^
Ni	5.41±0.23	6.73±0.25	1.53±0.07	1.10±0.05	0.68±0.01	0.72±0.01	5.0
Cu	1.42±0.06	0.41±0.01	0.18±0.01	0.09±0.00	0.12±0.01	0.04±0.00	---^b^
Zn	30.32±1.67	38.62±2.12	32.18±1.68	30.97±1.56	13.77±0.53	14.62±0.68	5.0
As	25.57±0.48	20.42±0.59	12.01±0.43	7.97±0.25	10.34±0.38	6.16±0.21	5.0
Cd	0.05±0.00	0.03±0.00	0.04±0.00	0.03±0.00	0.03±0.00	0.01±0.00	1.0
Pb	0.03±0.00	0.02±0.00	0.03±0.00	0.02±0.00	0.08±0.00	0.01±0.00	5.0

(1) a: USEPA, test methods for evaluating solid waste: physical/chemical methods (SW-846).

(2) b: Not titled.

**Table 4:** Concentrations of HMs in the combustion residual (mg/kg).

Samples	Cr	Mn	Ni	Cu	Zn	As	Cd	Pb
SS	156.50±5.56	1160.75±65.87	88.61±4.89	382.20±15.67	1448.33±70.45	62.36±2.56	3.35±0.08	111.87±3.32
SS-220-1h	200.90±6.78	1338.55±77.34	126.63±7.45	580.82±18.56	2026.21±68.62	62.58±2.03	4.62±0.10	168.50±5.25
SS-220-2h	182.66±6.68	1345.87±72.56	118.05±6.45	505.83±12.05	1950.73±55.24	44.65±1.89	4.66±0.11	147.99±3.98
SS-220-3h	187.80±8.56	1425.18±65.45	125.41±5.23	531.88±10.78	2010.28±69.46	41.61±1.01	4.63±0.09	152.88±4.23
SS-250-1h	184.07±10.56	1350.55±75.78	118.88±6.54	531.64±11.34	1964.51±51.67	41.36±1.56	4.55±0.13	152.55±4.01
SS-280-1h	219.89±11.45	1356.32±68.67	124.50±5.45	580.29±15.67	1972.96±75.34	38.39±1.12	4.69±0.08	153.34±4.23

**Table 5:** Residual rates of HMs in the combustion residual (%).

Samples	Cr	Mn	Ni	Cu	Zn	As	Cd	Pb
Raw SS	87.36	74.97	90.69	72.16	88.47	85.09	91.72	75.31
SS-220-1h	90.15	84.84	91.70	79.13	86.65	87.57	91.87	79.59
SS-220-2h	91.12	92.29	92.39	80.07	88.60	85.02	92.23	80.96
SS-220-3h	90.09	92.78	92.95	79.26	90.12	88.22	91.13	81.13
SS-250-1h	92.53	84.80	95.95	75.14	90.31	91.50	92.08	76.81
SS-280-1h	93.88	88.46	93.28	78.51	92.03	92.86	92.61	72.23

## Data Availability

The data used to support the findings of this study are available from the corresponding author upon request.
